# Interfacial Enhancement and Composite Manufacturing of Continuous Carbon-Fiber-Reinforced PA6T Composites via PrePA6T Ultrafine Powder

**DOI:** 10.3390/ma17071557

**Published:** 2024-03-28

**Authors:** Jiahong Yao, Zhao Wang, Jiacao Yang, Xiaojun Wang, Jie Yang

**Affiliations:** 1 College of Polymer Science and Engineering, Sichuan University, Chengdu 610064, China; yaojiahong@stu.scu.edu.cn (J.Y.); wangzhao2019@outlook.com (Z.W.); 2 Analytical and Testing Center, Sichuan University, Chengdu 610064, China; ppsf@scu.edu.cn; 3 State Key Laboratory of Polymer Materials Engineering, Sichuan University, Chengdu 610065, China

**Keywords:** carbon fiber, PA6T, sizing agent, thermoplastic composites, ultrafine powder

## Abstract

Semi-aromatic poly (hexamethylene terephthalamide) (PA6T) oligomer (prePA6T) ultrafine powder, with a diameter of <5 μm, was prepared as an emulsion sizing agent to improve the impregnation performance of CF/PA6T composites. The prePA6T hyperfine powder was acquired via the dissolution and precipitation “phase conversion” method, and the prePA6T emulsion sizing agent was acquired to continuously coat the CF bundle. The sized CF unidirectional tape was knitted into a fabric using the plain weave method, while the CF/PA6T laminated composites were obtained by laminating the plain weave fabrics with PA6T films. The interfacial shear strength (IFSS), tensile strength (TS), and interlaminar shear strength (ILSS) of prePA6T-modified CF/PA6T composites improved by 54.9%, 125.3%, and 120.9%, respectively. Compared with the commercial polyamide sizing agent product PA845H, the prePA6T sizing agent showed better interfacial properties at elevated temperatures, especially no TS loss at 75 °C. The SEM observations also indicated that the prePA6T emulsion has an excellent impregnation effect on CF, and the fracture mechanism shifted from adhesive failure mode to cohesive failure mode. In summary, a facile, heat-resistant, undamaged-to-fiber environmental coating process is proposed to continuously manufacture high-performance thermoplastic composites, which is quite promising in mass production.

## 1. Introduction

Carbon-fiber-reinforced thermoplastic composites (CFRTPs) have the advantages of good impact toughness, high damage tolerance, repairability, recyclability, and a short molding period compared with carbon-fiber-reinforced thermosetting composites (CFRTSs). Particularly, high-performance CFRTPs based on polyphenylene sulfide (PPS), polyether ether ketone (PEEK), polyetherimide (PEI), semi-aromatic poly (hexamethylene terephthalamide) (PA6T), etc., have great superiority in terms of heat resistance, mechanical properties, chemical stability, etc., which are widely applied in the aerospace, automotive, and military industries [1,2,3]. Semi-aromatic polyamide 6T (PA6T), the polycondensation product of terephthalic acid and hexanediamine, consists of rigid aromatic rings and flexible aliphatic chains, which integrate the excellent heat resistance and corrosion resistance of aromatic polyamides and the processibility and flowability of aliphatic ones. By virtue of its comprehensive properties, PA6T has extensive application prospects in the field of precision electronic and automobile parts.

CFRTPs have drawn great attention from researchers in both academia and industry due to their excellent comprehensive properties. The interface of fiber and resin is crucial in determining stress transfer. However, it is difficult to acquire a good interaction between fiber and resin due to the chemical inertia of CF. Accordingly, quite a few methods have been devised to promote interfacial interaction between resin matrix and CF by both physical and chemical means. Methods such as plasma treatment [4], oxidation treatment [5,6,7], high-energy radiation treatment [8,9], etc., are widely employed to introduce functional groups to activate the CF, although such CF modification methods require redundant treating procedures and would damage the CF’s intrinsic properties [4,10,11,12,13,14].

Among those existing fiber modification methods, sizing is a common, efficient, and low-cost way to treat carbon fiber, in which an even polymer coating is formed to improve the wettability, reactivity, adhesion, and abrasive resistance without damaging the CF chemical structure. However, the commercial sizing agents that are widely utilized now are mostly intended for CFRTSs [15,16], showing poor adhesion and stability [17] when it comes to thermoplastic composites, especially high-performance ones. Consequently, developing sizing agents specifically for high-performance CFRTPs with good compatibility and thermal resistance is of vital significance for special engineering thermoplastics. In addition, continuously processing and manufacturing continuous carbon-fiber-reinforced thermoplastic composites is indispensable in commercial mass production as continuous fibers in the matrix provide much stronger mechanical properties [18]. Hence, the sizing process must be facile and continuously operable. In our previous work [19], a PA6T oligomer was synthesized and deposited on a CF surface with the assistance of ultrasound, and the PA6T interfacial layer was coated on the CF surface via in situ polymerization, which significantly improved its interfacial properties. However, such ultrasound-assisted coating technology is not suitable for the continuous treatment of CF. On this basis, this paper aims to explore a facile and efficient technology to prepare continuous carbon-fiber-reinforced PA6T composites.

The high melt viscosity and weak interaction between resin and the fiber bundle [20,21,22,23] are the main points restricting the processing and overall performance of special engineering plastic composites. To solve such problems, a series of processing techniques were exploited, such as solution impregnation [21,22], pultrusion [24,25], powder impregnation [26,27], film lamination [28], fiber blending [29,30], etc. In fact, the insolubility of PA6T makes solution processing hard to implement. In addition, pultrusion requires sufficient dwell time in the customized die head for PA6T, which is at risk of thermal oxygen degradation or crosslinking [31,32,33,34]. Moreover, it is hard for film lamination processes to obtain a good impregnation state, which may result in many internal defects [28]. As for powder impregnation, the resin particles are dispersed in the fiber bundle and then melt to the molding, which evades the impregnation difficulty for high-viscosity melting. In modern powder impregnation processes, the size of the resin particles is usually over 50 μm, whereas the diameter of CF is around 5–7 μm. Research [26] has shown that the driving force for resin particles to penetrate the fiber bundle will rise much higher as the resin particle size increases. Moreover, it is hard to process polymers with high molecular weights into powders without damaging their intrinsic structures. Therefore, using resin particles with tiny sizes would be conducive to CF impregnation. Interfacial performance enhancement can be achieved via the in-situ polymerization of prePA6T.

Here, the dissolution and precipitation “phase conversion” method was put forward to acquire prePA6T hyperfine powder (diameter < 5 μm). The prePA6T powder was prepared into an oligomer emulsion sizing agent and continuously impregnated on a CF bundle using self-made spreading–impregnating equipment without an additional driving force, followed by lamination and hot-pressing with resin films after in situ polymerization to prepare continuous carbon-fiber-reinforced PA6T composites. On that basis, the effects of the solid content and polymerization degree on the micro- and macro-properties were discussed, and the discrepancy in the modification effect between the prePA6T emulsion sizing agent and the commercial product PA845H was compared. In summary, this article aims to explore an efficient, continuous, heat-resistant, undamaged-to-fiber coating technology for the preparation of high-performance thermoplastic composites with excellent interfacial performance and comprehensive properties.

## 2. Experimental Section

### 2.1. Materials

A PA6T (PA6T/6 = 50:50, random copolymer) oligomer with a degree of polymerization of around 4 was synthesized by a novel solution–solid phase one-pot polycondensation method according to our previous work [17,28]. The specific method is presented in the Supporting Information. PA6T (PA6T/6 = 50:50, random copolymer) resin was provided by Sanli Benzo New Materials Co., Ltd. (Qingdao, China). Carbon fiber (TC35-12K) (diameter: 7 μm) was purchased from Formosa Plastics Group (Taiwan, China). The PA6T oligomer (terephthalate-hexamethylamine salt) was synthesized in our laboratory. Concentrated sulfuric acid (98 wt%), N-methylpyrrolidone (NMP, AR, 99%), terephthalic acid (polymer grade), hexamethylenediamine (AR), caprolactam (CPL), and sodium dodecyl benzene sulfonate (SDBS, AR, 90%) were all acquired from Kelong Chemical Co., Ltd. (Chengdu, China) and used as received. Michelman Hydrosize TMPA845 fiber sizing agent (PA845H), a polyamide aqueous dispersion (solid content 22–24%), was supplied by Michelmen Chemical Trading Co., Ltd. (Shanghai, China), and polyethylene oxide (PEO, PFZ blue) was purchased from Sumitomo (Kyoto, Japan).

### 2.2. Sample Preparation for Micro-Bond Test

**Figure 1:**
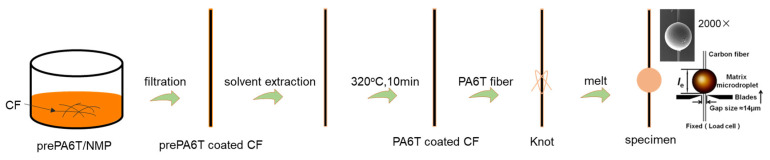
Schematic of single carbon fiber coating process.

As shown in Figure 1, the carbon fibers were immersed in the prePA6T/NMP solution at different concentrations (5 wt%, 10 wt%, 20 wt%, and 25 wt%) for 10 min. After filtering from the solution, the CFs were quickly placed in deionized water, so that the prePA6T precipitated on the CF surface and a uniform coating formed. Subsequently, the coated CFs were dried in an oven, followed by heating at 320 °C for 2, 5, 10, and 14 min to acquire PA6T sized CFs with different solution concentrations and reaction degrees. For comparison, CF was immersed in the commercial sizing agent PA845H for 10 min with the same post-treatments applied and was labeled as CF-PA845H. The microdroplets were prepared via the knotting method to characterize the micro-interfacial performance. After that, the PA6T fiber was knotted on the sized CF, and the microdroplet was acquired after being melted. The IFSS test was carried out on the microdroplet, and detailed experiment information can be seen in the Supplementary Materials.

For convenience of expression, the prePA6T-treated carbon fibers are named as CF-x-y min, where x and y denote the solution concentration and polymerization time, respectively.

### 2.3. Preparation of prePA6T Hyperfine Powder and Emulsion

PrePA6T with a degree of polymerization of around 4 was synthesized according to our previous work [19] and was then dissolved in NMP at 140 °C. The prePA6T hyperfine powder was acquired by the dissolution and precipitation “phase conversion” method, in which three techniques were applied to acquire prePA6T particles: rapid cooling precipitation in NMP, non-solvent precipitation in deionized water, and non-solvent precipitation in ethanol. For comparison, the prePA6T/NMP solution was sonicated to acquire oligomer powder. After washing and filtration, the powders were dispersed in deionized water to determinate the particle size and distribution so as to screen the technology with the smallest particle size and narrowest distribution.

To acquire a stable and uniform dispersion state, the prePA6T hyperfine powder was dispersed in deionized water with 1 wt‰ of SDBS and PEO added in. A series of dispersions with different solid concentrations were prepared (1 wt%, 2 wt%, 5 wt%, and 10 wt%).

### 2.4. Preparation of CF/PA6T Composites

**Figure 2:**
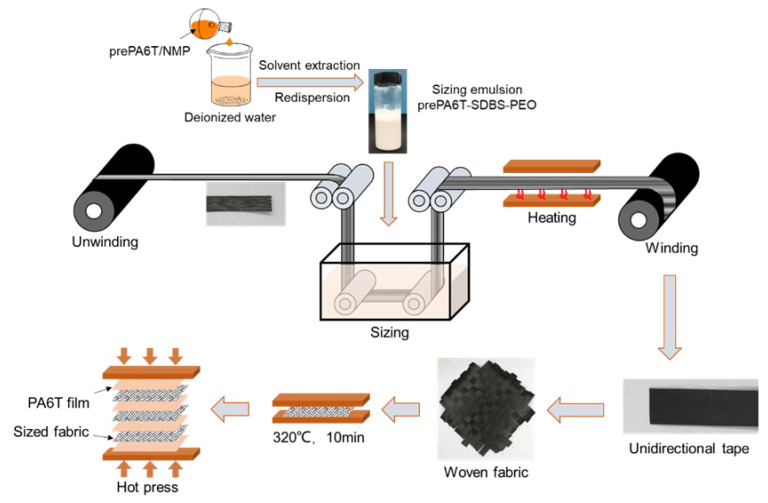
Schematic of continuous carbon fiber coating and CF/PA6T composite preparation.

As shown in Figure 2, a continuous preparation route of CF/PA6T composites was exploited to acquire tensile and ILSS test specimens. The CF unidirectional tape was prepared by impregnating the continuous CF bundles with the prePA6T emulsion sizing agent and the commercial product PA845H. PA6T film was prepared by hot-pressing the PA6T resin at 320 °C with an applied pressure of 2.5 MPa. Meanwhile, the unidirectional tape was knitted into fabric by plain weave method.

Next, the plain weave fabric was hot-pressed at 320 °C for 2 min, 5 min, 10 min, and 14 min to explore the effect of polymerization degree on the composite performance. PA6T-laminated composites were prepared by laminating the PA6T resin films and sized fabrics. Based on the polymerization time, the unidirectional tape with a coating concentration of 10 wt% was named as CF-10′-z min, where 10′ represents the coating concentration and z represents the polymerization time.

### 2.5. Characterization

The intrinsic viscosity, particle size and distribution, surface morphology, heat resistance, and macro- and micro-mechanical properties were characterized. The experimental details can be seen in the Supplementary Materials.

## 3. Results and Discussion

### 3.1. The Morphology and IFSS of Sized Single CF

**Figure 3:**
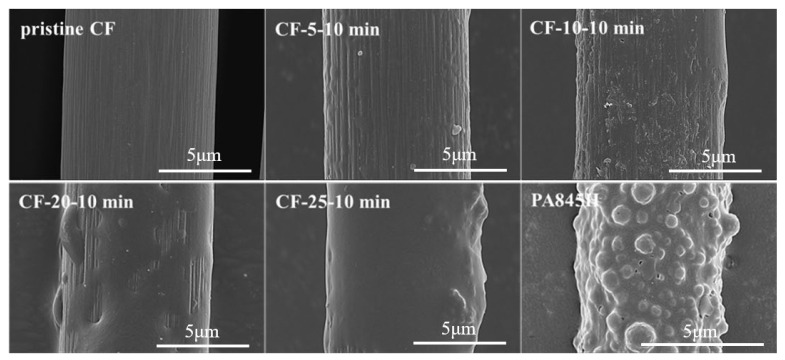
Fiber morphology with different concentrations of PA6T oligomer solution coated.

The surface morphology of the sized CFs was inspected by the SEM test. As shown in Figure 3, few resins could be found on the fiber surface at 5 wt% concentration. As the coating concentration increased, the coated resin layer thickened. At 25 wt% concentration, a complete coat formed on the CF surface and grooves, where in situ polymerization occurred to enhance the stress transfer efficiency. For comparison, the modification effect of the commercial aliphatic PA sizing agent PA845H was also discussed. As we can see, the polyamide nanoparticles aggregated on the surface of the PA845H-treated fiber with the assistance of the film-forming agent, and a compact coating formed so as to bridge the resin matrix and CF. However, additives in PA845H such as a film-forming agent would worsen the heat resistance and overall performance of composites.

**Figure 4:**
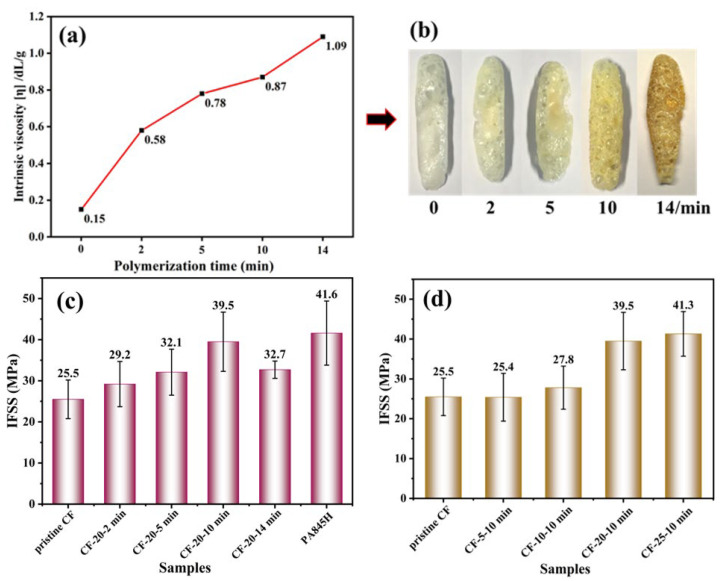
The intrinsic viscosity (**a**) and surface morphology (**b**) of PA6T coating with different polymerization times, and the IFSS of CF/PA6T composites with different polymerization times (**c**) and different concentrations of PA6T oligomer coating (**d**).

As we can see in Figure 4a, the intrinsic viscosity increased as the polymerization time lengthened due to the growing molecular weight. Interestingly, the viscosity rose rapidly in the early polymerization stage as more active terminal groups existed. After 10 min of polymerization, the [η] rose to 0.87 dL/g, which closely resembled the matrix resin’s value of 0.89 dL/g. Figure 4b shows that the resin turned yellow as the polymerization time increased, indicating that the resin underwent slight thermal and oxygen degradation. When the reaction time increased to 14 min, the resin color deepened and the intrinsic viscosity increased rapidly to 1.09 dL/g, indicating the forming of a cross-linked structure.

Some research has been conducted on the effect of sizing agent molecular weight on the interfacial performance of thermosetting plastic composites [35,36,37,38]. It was demonstrated that a thermosetting sizing agent with a high molecular weight would make the molecular chain move hard, resulting in a weak interface with the bulk resin matrix. Conversely, low-molecular-weight ones have more active groups, markedly enhancing the wettability of the fiber surface and rendering a stronger interaction. However, there are few reports on thermoplastic sizing agents. It can be seen in Figure 4c that the IFSS gradually increased as the polymerization degree increased when the polymerization time was less than 14 min. It could be explained that the linear molecular structure of prePA6T allowed for better mobility, despite having a higher molecular weight. In addition, the long molecular chains were more likely to tangle with the matrix resins, and more amide bonds provided stronger hydrogen bonding, so the IFSS performance enhanced as the polymerization time increased. Nevertheless, a long polymerization time at high temperatures would intensify the thermal oxygen degradation of the coating layer [39,40], thus bringing about a decrease in IFSS value at 14 min polymerization time. In conclusion, the IFSS of CF-20-10 min increased from 25.5 MPa initially to 39.5 MPa with an increase rate of 55%. This improvement is comparable to that of PA845H, which saw an increase of 63% to 41.6 MPa.

The micro IFSS value did not fluctuate much at low coating concentrations, which indicated that the polymer particles were too dispersed and diluted at a low content to form a complete coating over the fiber. Therefore, the enhancement effect on the interfacial performance was not notable enough, which was consistent with the SEM images of the surface morphology in Figure 3. At 20 wt% concentration, a stable and interconnected coating formed, which gave rise to a remarkable improvement of 54.9% in the IFSS. After that, the IFSS did not increase much at 25 wt% concentration as a complete coating had formed.

**Figure 5:**
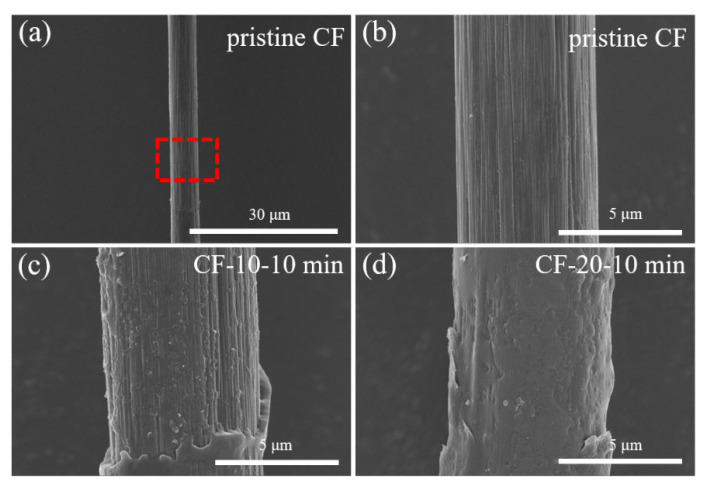
Fiber morphology after debonding with different concentrations of PA6T oligomer coated: (**a**) pristine CF; (**b**) magnification of highlighted part of (**a**) pristine CF; (**c**) CF-10-10 min; (**d**) CF-20-10 min.

The adhesiveness between a resin and a fiber can be determined by the fiber morphology after debonding. As shown in Figure 5, the surface of the debonded pristine CF was smooth and no residual resin existed in the grooves, indicating a poor interaction between the resin matrix and pristine CF. By contrast, residual resin could be observed on the surface and grooves of sized CFs after debonding, which verified that the low-viscosity oligomer had better impregnation on the CF. After in situ polymerization, a stable and uniform polymer coating formed on the CF surface, effectively bridging the fiber and resin matrix and improving the IFSS of the composites.

### 3.2. Interfacial Properties at Elevated Temperatures

**Figure 6:**
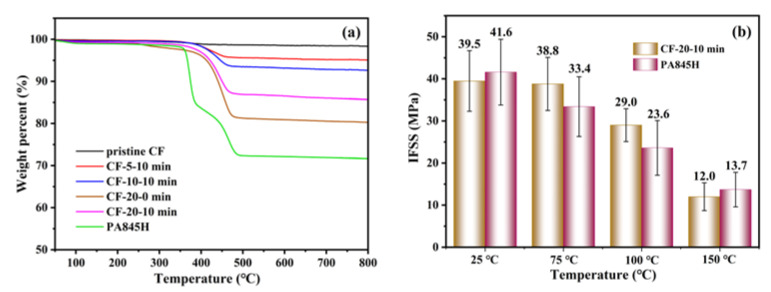
The TGA curves of PA6T oligomer solution-coated CF (**a**); the IFSS comparison between PA6T oligomer- and PA845H-coated CF/PA6T composites under elevated testing temperatures (**b**).

High-performance CFRTPs are usually utilized in harsh service environments such as high temperatures, so heat resistance is vital for composites. Therefore, TGA and the micro-bond test under elevated temperatures were employed. The TGA curves in Figure 6 show that the thermal decomposition temperatures of the prePA6T-modified CF were over 400 °C, whereas the PA845H-modified CF had a lower thermal decomposition temperature and char yield. There are two stages of losing weight for PA845H. The first stage is at around 350 °C, which is attributed to the decomposition of dispersing agent and film-forming agent. The second stage is at over 400 °C, which results from the decomposition of the aliphatic polyamide backbone. There were two main reasons for this discrepancy in thermal stability. Firstly, the rigid benzene rings in the PA6T backbone enhanced the thermal tolerance, while the aliphatic polyamide in PA845H with more flexibility reduced the heat resistance. Secondly, the heat-labile additives in PA845H contributed to the approximately 15% weight loss at around 350 °C.

Comparing the IFSS of CF-20-10 min and CF-PA845H at elevated temperatures, the IFSS decreased steadily as the temperature increased. It is believed that the grasping force of the resin microdroplet on CF weakened due to the discrepancy in thermal expansion coefficient between the resin and CF. Additionally, the shrinking static friction force, originating from the resin grasping force on fiber in the radial direction, accounted for the major loss in IFSS [41]. As shown in Figure 6, the IFSS of CF-20-10 min at 75 °C decreased by merely 0.7 MPa compared to 25 °C, while the IFSS of CF-PA845H dropped by 19.7%, which resulted from the heat resistance discrepancy of the two sizing agents. As discussed before, there are plenty of additives with poor heat resistance in PA845H, and the primary component of PA845H is aliphatic polyamide, whose T_g_ is 50–55 °C [42]. As the temperature was elevated at 75 °C (above the T_g_ of aliphatic polyamide), the grasping force weakened notably due to the remarkably increasing thermal expansion coefficient discrepancy between the fiber and resin. However, the semi-aromatic polyamide PA6T has a T_g_ of 85 °C, which shows a lower thermal expansion coefficient at 75 °C. Thus, the in situ polymerization-modified PA6T coating has a better interfacial modification effect at elevated temperatures than PA845H.

### 3.3. PrePA6T Particle Size Distribution and Emulsion Preparation

**Figure 7:**
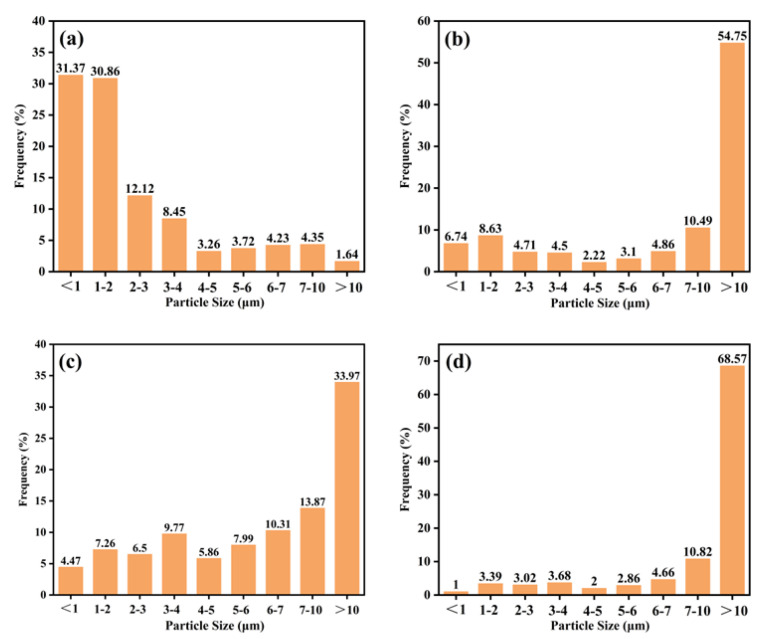
The oligomer particle size and distribution obtained via phase separation of PA6T oligomer solution by (**a**) non–solvent precipitation in deionized water (**b**) and non–solvent precipitation in ethanol; (**c**) rapid cooling precipitation in NMP; (**d**) sonication in NMP.

Considering the complex post-treatment techniques and large solvent consumption in the solution coating process, it is challenging to implement the coating of continuous fiber, so it was necessary to prepare prePA6T powder as a stable emulsion slurry. The dissolution and precipitation “phase conversion” method was exploited to prepare polymer micro-particles [43]. In this method, the solutes are precipitated from the solvent by rapidly cooling or adding non-solvent to acquire particles with micron or nanoscale size, which is a common way to prepare inorganic nanomaterials. In this study, the best precipitation condition was explored, and the oligomer particle size and distribution are shown in Figure 7. The results showed that deionized water is the best candidate for the precipitant. According to the Flory–Huggins theory, the capacity for a polymer solution to precipitate depends on the interaction parameter between non-solvent and solvent. It was found that the interaction between H_2_O and NMP is stronger than that between ethanol and NMP. When the oligomer/NMP solution was poured into the water, the intense interaction broke the thermodynamic equilibrium, and the nucleation sites were too numerous to adhere to each other, contributing to the tiny particle size.

Based on this, the surfactant SDBS and the thickener PEO were added to keep the emulsion stable. It was shown that the emulsion has stability as good as commercial PA845H, and no apparent sediment was observed after one month.

### 3.4. Morphologies of Emulsion-Coated CF Unidirectional Tapes

**Figure 8:**
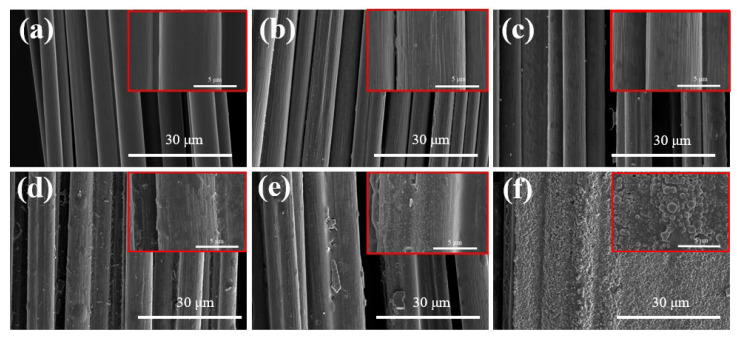
The CF bundle morphology with different concentrations of PA6T oligomer coated: (**a**) pristine CF; (**b**) CF-1′-10 min; (**c**) CF-2′-10 min; (**d**) CF-5′-10 min; (**e**) CF-10′-10 min; (**f**) PA845H (higher magnification images were marked with red box).

The morphologies of the coated CF unidirectional tapes are shown in Figure 8. Similar with Figure 3, little resin could be found on the surface of CF-1′-10 min and CF-2′-10 min. At 10 wt% concentration, the CF was entirely wrapped by resin, and the interspaces between CFs were adequately filled. Comparatively, it was hard to distinguish the single filament for CF unidirectional tape coated by PA845H with a higher solid content, suggesting that PA845H had an excellent coating and film-forming performance.

### 3.5. Mechanical Properties of Fabric PA6T/CF Composites

**Figure 9:**
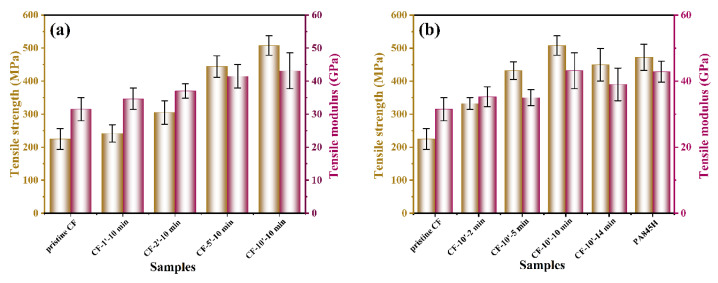
(**a**) The tensile strength of CF/PA6T composites with different concentrations of PA6T oligomer modification; (**b**) the tensile strength of CF/PA6T composites with different polymerization times of PA6T oligomer coating.

**Figure 10:**
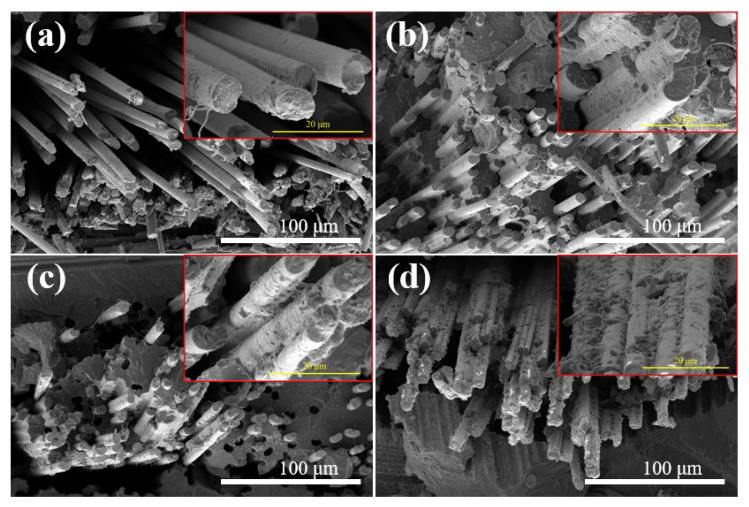
The fracture morphology of CF/PA6T composites: (**a**) pristine CF; (**b**) CF-5′-10 min; (**c**) CF-10′-10 min; (**d**) PA845H (higher magnification images were marked with red box).

Tensile performance is an important indicator to evaluate the macro-mechanical property of fabric CF/PA6T composites, and the results are displayed in Figure 9 and Tables S1 and S2. The pristine CF reinforced composites exhibited a tensile strength of 225.4 MPa, which indicated a poor interaction between the CF and resin matrix, in accordance with the IFSS and SEM results. The higher coating concentration enhanced the tensile strength distinctly, which is attributed to a better impregnation state without internal defects, thus improving the stress transfer effect. According to the fracture morphology of the CF/PA6T composites presented in Figure 10, as the coating concentration increased, the pull-out CFs displayed a rougher surface, and residual resin could be found on the surface. As shown in Figure 9b, the variation trend of tensile performance was not completely positively related with the polymerization time. As elaborated before, when the polymerization time exceeded 10 min, the mechanical performance deteriorated due to thermal oxygen degradation. It is worth nothing that although PA845H-coated CF had the best IFSS, the tensile performance was not optimal, which is attributed to the additives in the coating layer resulting in more defects at the interface.

**Figure 11:**
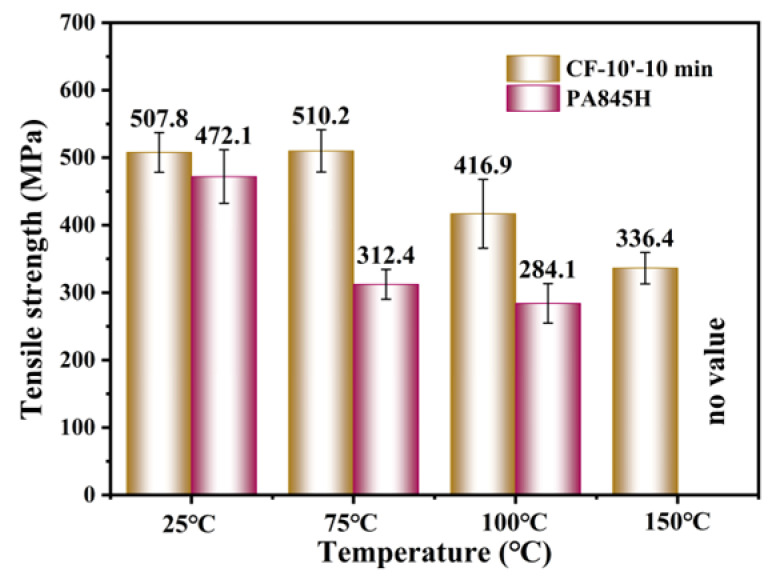
The tensile strength of CF/PA6T composites under elevated testing temperatures.

To verify the high-temperature mechanical performance, a series of tensile tests of composites were carried out under elevated temperatures. The results in Figure 11 are in line with the IFSS results, as CF-10′-10 min had almost no decline when the temperature was elevated at 75 °C, while the PA845H-modified composite showed a sharper decline. Consistent with the IFSS results at elevated temperatures, it was verified again that the prePA6T-modified composites have good high-temperature mechanical properties, and there was no loss of strength under T_g_ (85 °C), which is superior to the composites modified by PA845H. For CF-PA845H, tensile strength could not be acquired at 150 °C due to the softening of PA845H. In brief, it is shown, thus, that the prePA6T emulsion has not only an equivalent interfacial modification performance but also better mechanical properties at elevated temperature, which is more adaptable to complicated and rigorous application scenarios.

The tensile properties of the carbon fiber fabric-reinforced polyamide composites prepared by different processing methods are listed in Table 1. Compared with the direct forming of fiber fabric, a novel process of hot-pressing film with in situ polymerized prePA6T ultrafine powder impregnating the fiber bundle is innovated in this research system. In our work, better tensile properties could be achieved with a lower fiber content, which was mainly due to to the better infiltration and penetration of the low-viscosity prePA6T hyperfine powder and the stronger bonding force with CF after in situ polymerization. Moreover, the 3D printed CF/PA12 composite had higher tensile strength for the higher fiber orientation in the unidirectional composite system.

### 3.6. ILSS of Fabric Laminated Composites

**Figure 12:**
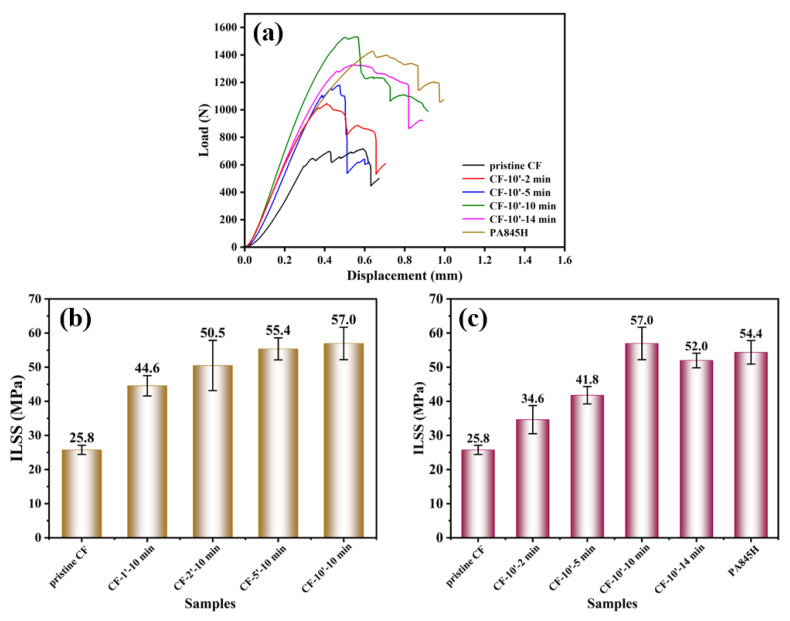
The interlaminar shear property of CF/PA6T composites: (**a**) force–displacement curve; the ILSS of composites with different (**b**) coating concentrations and (**c**) polymerization times.

**Figure 13:**
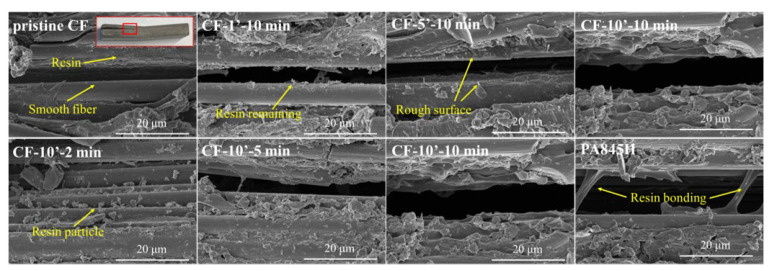
The interlaminar fracture (crack propagation) morphology of composites (the observation spot was marked with red box).

Interlaminar failure is the primary failure form of laminated composites, so ILSS (Interlaminar Shear Strength) is the most critical performance criterion for evaluating this, as well as the most direct performance parameter that reflects the macro fiber–resin interfacial properties of composite materials [47]. According to the IFSS results, the micro IFSS value rose with the polymerization degree increasing, which aligned with the macro ILSS variation tendency shown in Figure 12. Moreover, the ILSS of CF-10′-10 min was elevated from 25.8 MPa in the pristine state to 57.0 MPa, an increase of 120.9%. The result verifies that the higher molecular weight of the interfacial layer brings high mechanical strength, strong hydrogen bonding, and more molecular chain entanglement, which effectively prompts the improved interfacial performance. The SEM images in Figure 13 also indicate that the interlaminar fracture morphology changed from finely divided resin particles (CF-10′-2 min) to tearing films (CF-10′-10 min). However, with the weakening of the thermal oxygen aging reaction on the interfacial layer, the ILSS of CF-10′-14 min deteriorated.

In addition, although the PA845H-modified CF/PA6T composite had the highest micro IFSS, its macro ILSS was still lower than that of CF-10′-10 min, probably resulting from other components in the slurry having introduced plentiful interfaces and defects, destroying the uniformity and integrality of the matrix.

Figure 13 presents the cracks in the delaminated composites to evaluate the interlayer reaction. In Figure 13, the pristine CF shows a smooth surface, indicating poor interaction between the CF and resin matrix. On increasing the concentration of prePA6T emulsion and the polymerization time, more remaining resin could be observed on the CF, showing the enhanced interaction. Interestingly, the resin bonding phenomenon could only be found in the interlaminar fracture of the PA845H-modified composite, indicating that other components in the PA845H slurry may play a role in improving the interlayer toughness. The resin bonding slows down the crack propagation speed and enhances the interlayer crack toughness, which can also be confirmed by the load–displacement curve in Figure 12.

## 4. Conclusions

In this paper, prePA6T hyperfine powder with a diameter of less than 5 μm and narrow size distribution was prepared via the dissolution and precipitation “phase conversion” method, and a stable prePA6T sizing agent was acquired. Remarkably, the prePA6T sizing agent has excellent heat resistance and interfacial performance under elevated temperatures. The effects of polymerization time and emulsion concentration on fiber surface morphologies, IFSS, ILSS, and tensile performance were also discussed.

At a low coating concentration, the IFSS did not vary much. At 20 wt% concentration, the IFSS increased rapidly by 55% as a uniform coating formed and the entanglement and hydrogen bonding enhanced. The higher concentration than this did not improve the IFSS much. Further, a long polymerization time (320 °C, 14 min) would lead to thermal oxidation aging and degrade the IFSS. Compared with PA845H, the PA6T coating exhibited better mechanical performance at an elevated temperature. Concretely, PA6T showed no IFSS loss under 75 °C.

The prePA6T ultrafine powder prepared by the “phase conversion” method had a tiny and narrow particle size distribution. The emulsion sizing agent prepared using the prePA6T oligomer had good penetration and impregnation effects on CF bundles. As the emulsion concentration increased to 10 wt%, the tensile strength of the fabric laminated composite rose to 507.8 MPa, achieving a higher tensile strength than the aliphatic PA composites prepared by injection molding at a low fiber fraction. Moreover, increasing the polymerization degree enhanced the interfacial interaction, thus improving the tensile strength. Notably, the prePA6T-modified composites had no tensile strength loss at 75 °C, and the tensile strength at 100 °C remained over 80%. However, CF-PA845H had a poor mechanical performance at elevated temperatures due to poor heat resistance and additional components.

The ILSS was improved with increasing the emulsion concentration and polymerization. A higher emulsion concentration (10 wt%) and proper polymerization degree (320 °C-10 min) endowed better interaction between CF and resin. CF-10′-10 min had the best TS and ILSS, having improved by 125.3% and 120.9%, while the CF-PA845H improved by 109.4% and 120.9%. Further, the additional component in PA845H led to the existence of resin bonding, enhancing the interlayer crack toughness.

In summary, a facile, continuous, heat-resistant, undamaged-to-fiber, and environmental coating technology was exploited to manufacture continuous fiber-reinforced PA6T composites. The CF/PA6T interfacial performance was efficiently improved, and high-performance thermoplastic composites with exceptional properties were prepared successfully.

## Figures and Tables

**Table 1:** The tensile strength of CF/PA composites obtained via different preparation techniques.

Samples	CF/PA6 [44]	CF(40 vol%)/PA66 [45]	CF(31.9vol%)/PA12 [46]	CF(36.2% vol%)/PA6T this Work
Processing method	T-RTM (fabric)	Interfacial polymerization (fabric)	3D printing (UD)	Powder + compression molding (fabric)
Tensile strength (MPa)	431.2	408	530.1	507.8
Tensile modulus (GPa)	34.7	35.4	54.8	43.1

## Data Availability

All the data are available in the paper.

## Supplementary Materials

The following supporting information can be downloaded at: https://www.mdpi.com/article/10.3390/ma17071557/s1, Figure S1: Synthesis of PA6T, (PA6T/6 = 50:50, random copolymer; Figure S2: The tensile specimen of continuous CF/PA6T composite; Figure S3: Interlaminar shear test of continuous CF/PA6T composite; Table S1: The tensile strength of CF/PA6T composite with different emulsion concentration coated; Table S2: The tensile strength of CF/PA6T composite with different polymerization time.
