# Supplementary material for: Interfacial Enhancement and Composite Manufacturing of Continuous Carbon-Fiber-Reinforced PA6T Composites via PrePA6T Ultrafine Powder
Source: Materials (Basel). 2024 Mar 28;17(7):1557. doi: 10.3390/ma17071557 (PMC11012839; doi:10.3390/ma17071557)
Supplement: Supplementary file 1 [file materials-17-01557-s001.zip › materials-2908519-supplementary.pdf]

Supporting Information

Interfacial Enhancement and composite manufacturing of continuous carbon fiber reinforced PA6T composites via PrePA6T ultrafine powder

Jiahong Yao [1], Zhao Wang [1], Jiacao Yang [2,*], Xiaojun Wang [2,*] and Jie Yang [2,3]

1 College of Polymer Science and Engineering, Sichuan University, Chengdu 610064, China; yaojiahong@stu.scu.edu.cn (J. Y.); wangzhao2019@outlook.com (Z. W.)
2 Analytical and Testing Center, Sichuan University, Chengdu 610064, China; jiacaoyang@outlook.com (J. Y.); wangxj@scu.edu.cn (X. W.); ppsf@scu.edu.cn (J. Y.)
3 State Key Laboratory of Polymer Materials Engineering, Sichuan University, Chengdu 610065, China; ppsf@scu.edu.cn (J. Y.)
* Correspondence: jiacaoyang@outlook.com (J. Y.); wangxj@scu.edu.cn (X. W.)

Synthesis of prePA6T

The PA6T oligomer (prePA6T) was synthesized by the solution-phase one-pot polycondensation method, and 50% amount of flexible aliphatic PA6 segments were introduced into the aromatic backbone to improve the processibility. Concretely, certain amount of caprolactam (CPL), hexamethylenediamine and terephthalic acid were added in the sealed reactor at the pressure of 2.3MPa with N_2 atmosphere. For convenience of expression, the copolymer of PA6T and PA6 was named as PA6T.

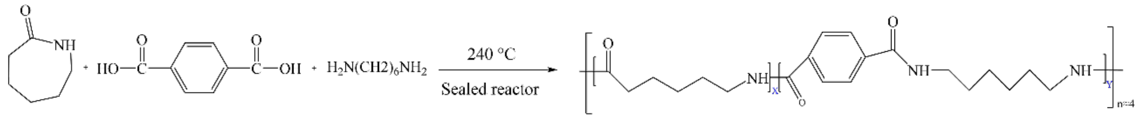

Figure S1 Synthesis of PA6T, (PA6T/6 = 50:50, random copolymer

Characterization

Viscosity test

The viscosity measurement was carried out on a ubbelohde viscometer, with sulfuric acid as solvent， and the concentration for PA6T is 0.5g/dL. The intrinsic viscosity can be calculated by the Solomon Ciuta equation:

$$[\eta] = \frac{\sqrt{2(\eta_{sp} - ln\eta_r)}}{C} \tag{1}$$

Where $\eta_{sp}= \eta/\eta_0-1$, $\eta_r= \eta/\eta_0$, and C represents the concentration of polymer solution. As η is in direct proportion to the flowing time through capillary, η_r can be acquired by equation:

$$\eta_r = \frac{t}{t_0} \tag{2}$$

where t and t_0 denote the flowing time through the capillary, respectively.

SEM observation

The scanning electron microscope (Nova NanoSEM450, USA) was applied to observe the surface morphologies of the carbon fibers and fracture features of composites.

Particle size and distribution analysis

The prePA6T particle size and distribution were measured with the wet and dry laser particle size analyzer (HELOS SR, Germany).

Microbond test

The interfacial shear strength (IFSS) was performed on the microbond test apparatus, through which PA6T droplets were pulled out from the single fiber by two fixed blades at a speed of 0.02 mm/s, and the debonding load was accurately recorded till the maximum pull-out force was reached. The apparent IFSS (τ_{app}) could be calculated through the following equation:

$$\tau_{app} = \frac{F_{max}}{\pi d_f l_e} \tag{3}$$

Where F_{max} is the maximum pull-out force during the process, d_f and l_e denote fiber diameter and embedded length in the droplet, respectively. At least 30 values should be successfully measured to acquire the average value.

Tensile and ILSS test

The CF/PA6T laminated composites was tailored to a size of 120 mm×10 mm×1 mm for tensile test, with the glass fiber reinforced epoxy sheets as the reinforcing sheet to avoid the stress concentration at the end of specimens. The tensile test was carried out with the electromechanical universal testing machine (MTS Exceed E45). For elevated temperature tests, the experiment was conducted at 25 ºC (room temperature), 75 ºC, 100 ºC, 150 ºC, respectively, at a tensile speed of 2 mm/min tensile. The tensile strength and Young's modulus were recorded, and at least 5 specimens should be tested to obtain the average value.

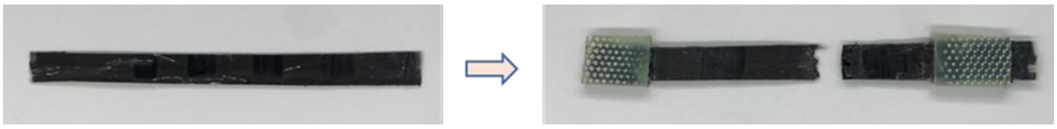

Figure S2 The tensile specimen of continuous CF/PA6T composite

For ILSS test, the specimen was tailored to a size of 15 mm×5 mm×2.5 mm. According to ASTM D 2344, the ILSS was carried out as shown in Figure S2 at a loading speed of 1 mm/min, and the ILSS was calculated by the following equation:

$$ILSS = 0.75 * \frac{P}{bh} \quad (4)$$

Where P is the maximum load, b and h represent the thickness and width of specimen, respectively. Similarly, 5 specimens are required at least to obtain the average value of ILSS.

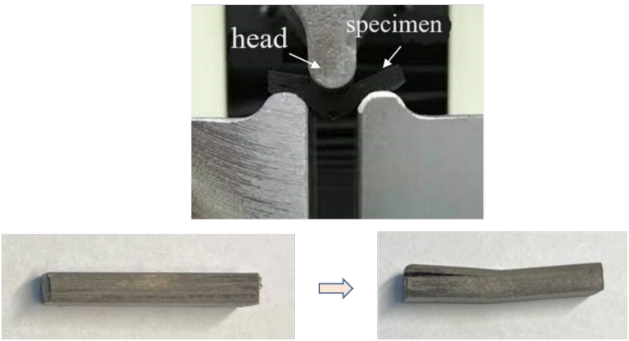

Figure S3 Interlaminar shear test of continuous CF/PA6T composite

Tensile strength of CF/PA6T composites

Table S1 The tensile strength of CF/PA6T composite with different emulsion concentration coated

Samples	Pristine CF	CF-1'-10 min	CF-2'-10 min	CF-5'-10 min	CF-10'-10 min	PA845H
Vol%	36.5	36.2	36.0	35.1	36.2	36.6
Tensile strength (MPa)	225.4(±31.3)	241.9(±26.5)	305.1(±35.6)	444.6(±32.4)	507.8(±29.3)	472.1 (±39.7)
Tensile modulus (GPa)	31.5(±3.5)	34.6(±3.2)	37.0(±2.2)	41.5(±3.6)	43.1(±5.4)	42.9(±4.2)

Table S2 The tensile strength of CF/PA6T composite with different polymerization time

Samples	Pristine CF	CF-10'-2min	CF-10'-5 min	CF-10'-10 min	CF-10'-14 min	PA845H
Vol%	36.5	36.4	36.5	36.2	36.3	36.6
Tensile strength (MPa)	225.4(±31.3)	241.9(±26.5)	305.1(±35.6)	444.6(±32.4)	507.8(±29.3)	472.1 (±39.7)
Tensile modulus (GPa)	31.5(±3.5)	34.6(±3.2)	37.0(±2.2)	41.5(±3.6)	43.1(±5.4)	42.9(±4.2)
